# Knowledge of Hyperemic Myocardial Blood Flow in Healthy Subjects Helps Identify Myocardial Ischemia in Patients With Coronary Artery Disease

**DOI:** 10.3389/fcvm.2022.817911

**Published:** 2022-02-03

**Authors:** Lijuan Lyu, Jichen Pan, Dumin Li, Xinhao Li, Wei Yang, Mei Dong, Chenghu Guo, Peixin Lin, Yeming Han, Yongfeng Liang, Junyan Sun, Dexin Yu, Pengfei Zhang, Mei Zhang

**Affiliations:** ^1^The Key Laboratory of Cardiovascular Remodeling and Function Research, The State and Shandong Province Joint Key Laboratory of Translational Cardiovascular Medicine, Department of Cardiology, Chinese Ministry of Education, Chinese National Health Commission and Chinese Academy of Medical Sciences, Qilu Hospital, Cheeloo College of Medicine, Shandong University, Jinan, China; ^2^Department of Radiology, Qilu Hospital, Cheeloo College of Medicine, Shandong University, Jinan, China

**Keywords:** computed tomography myocardial perfusion imaging, myocardial blood flow, fractional flow reserve, myocardial ischemia, coronary artery disease

## Abstract

**Backgrounds:**

Dynamic CT myocardial perfusion imaging (CT-MPI) allows absolute quantification of myocardial blood flow (MBF). Although appealing, CT-MPI has not yet been widely applied in clinical practice, partly due to our relatively limited knowledge of CT-MPI. Knowledge of distribution and variability of MBF in healthy subjects helps in recognition of physiological and pathological states of coronary artery disease (CAD).

**Objectives:**

To describe the distribution and normal range of hyperemic MBF in healthy subjects obtained by dynamic CT-MPI and validate whether it can accurately identify functional myocardial ischemia when the cut-off value of hyperemia MBF is set to the lower limit of the normal range.

**Materials and Methods:**

Fifty-one healthy volunteers (age, 38 ± 12 years; 15 men) were prospectively recruited. Eighty patients (age, 58 ± 10 years; 55 men) with suspected or known CAD who underwent interventional coronary angiography (ICA) examinations were retrospectively recruited. Comprehensive CCTA + dynamic CT-MPI protocol was performed by the third – generation dual-source CT scanner. Invasive fractional flow reserve (FFR) measurements were performed in vessels with 30–90% diameter reduction. ICA/FFR was used as the reference standard for diagnosing functional ischemia. The normal range for the hyperemic MBF were defined as the mean ± 1.96 SD. The cut-off value of hyperemic MBF was set to the lower limit of the normal range.

**Results:**

The global hyperemic MBF were 164 ± 24 ml/100 ml/min and 123 ± 26 ml/100 ml/min for healthy participants and patients. The normal range of the hyperemic MBF was 116–211 ml/100 ml/min. Of vessels with an ICA/FFR result (*n* = 198), 67 (34%) were functionally significant. In the per-vessel analysis, an MBF cutoff value of <116 ml/100 ml/min can identify myocardial ischemia with a diagnostic accuracy, sensitivity, specificity, positive predictive value, and negative predictive value of 85.9% (170/198), 91.0% (61/67), 83.2 % (109/131), 73.5% (61/83), and 94.8% (109/115). CT-MPI showed good consistency with ICA/FFR in diagnosing functional ischemia, with a Cohen's kappa statistic of 0.7016 (95%CI, 0.6009 – 0.8023).

**Conclusion:**

Recognizing hyperemic MBF in healthy subjects helps better understand myocardial ischemia in CAD patients.

## Introduction

Coronary computed tomography angiography (CCTA) has become a reliable diagnostic technique to evaluate coronary artery disease (CAD) with high sensitivity and a negative predictive value (1–3). However, CCTA provides only anatomic information and tends to overestimate stenosis severity (2–4) and is limited in its ability to diagnose myocardial ischemia. Thus, CT myocardial perfusion imaging (CT-MPI) has been developed for myocardial blood flow (MBF) evaluation (5, 6). Combination of CCTA with CT-MPI can merge anatomical and physiological information, and provide a comprehensive interpretation of CAD.

Although appealing, CT-MPI has not yet been widely applied in clinical practice, partly due to our relatively limited knowledge of CT-MPI. In contrast to static perfusion imaging protocol, dynamic CT-MPI allows quantitative assessment of MBF. Beyond the assessment of the physiologic importance of a known epicardial stenosis, absolute MBF quantification offers potential advantages in identifying balanced ischemia and detection of microvascular disease. However, it is difficult to definitively classify quantitative MBF as normal or abnormal based on available knowledge. Understanding the normal blood flow distribution of the myocardial may have a role in the further delineation of these disease state. Previous publications with positron emission computed tomography (PET) (7, 8) and magnetic resonance imaging (MRI) (9) have suggested absolute MBF values for normal participants and thresholds for discriminating ischemic myocardial disease. However, previous studies have shown that MBF values vary with different imaging modalities, and that CT underestimates the stress MBF of normal myocardia compared with PET (5, 7, 8, 10–13). Therefore, it is necessary to establish the normal range of MBF through dynamic CT-MPI. To the best of our knowledge, only a few small-sample studies have reported the value ranges of MBF in normal subjects based on CT-MPI (11, 13). However, the results were inconsistent. The studies mentioned above were both performed on previous generation CT scanners but were conducted using different stressor. Apart from true variability, variability in MBF measurements can be affected by many factors, such as heterogeneity of included patients, scanner, image acquisition and reconstruction, postprocessing software, and calculation algorithms. Nonetheless, as revealed by previous studies, scanner- and software-specific normal values of MBF is still of clinical importance to guide image interpretation of dynamic CT-MPI (14). To date, there is no report on the normal range of the hyperemic MBF in healthy subjects using a third – generation dual-source CT (DSCT) scanner.

In this study, we aimed to explore the normal range of the hyperemic MBF from dynamic CT-MPI in healthy individuals using the third – generation DSCT scanner. Based on the determined range of the hyperemic MBF, we also aimed to validate the diagnostic value of the lower limit of normal range of MBF for diagnosing myocardial ischemia defined by invasive coronary angiography (ICA)/fractional flow reserve (FFR).

## Materials and Methods

### Study Population

Fifty-one healthy volunteers [age, 38 ± 12 years (22 – 59 years); 15 men] that completed adenosine-stressed dynamic CT-MPI between August 2019 and January 2021 were prospectively recruited by public advertising or from hospital staff. The inclusion criteria required that all volunteers be aged between 18 and 60 years, with normal blood pressure, normal blood lipid level, normal blood sugar level, normal effort tolerance, normal results upon physical examination and normal electrocardiogram, and transthoracic echocardiogram examination. The exclusion criteria were as follows: (a) any medical history of heart disease; (b) risk factors for coronary artery disease; (c) angina pectoris or equivalent symptoms; (d) contraindications to adenosine or iodine contrast media; (e) any history of endocrine diseases, respiratory diseases, connective tissue disease, and anemia and severe abnormal liver function; (f) history of alcoholism; (g) pregnancy; (h) coronary with any stenosis by CCTA.

**Figure 1:**
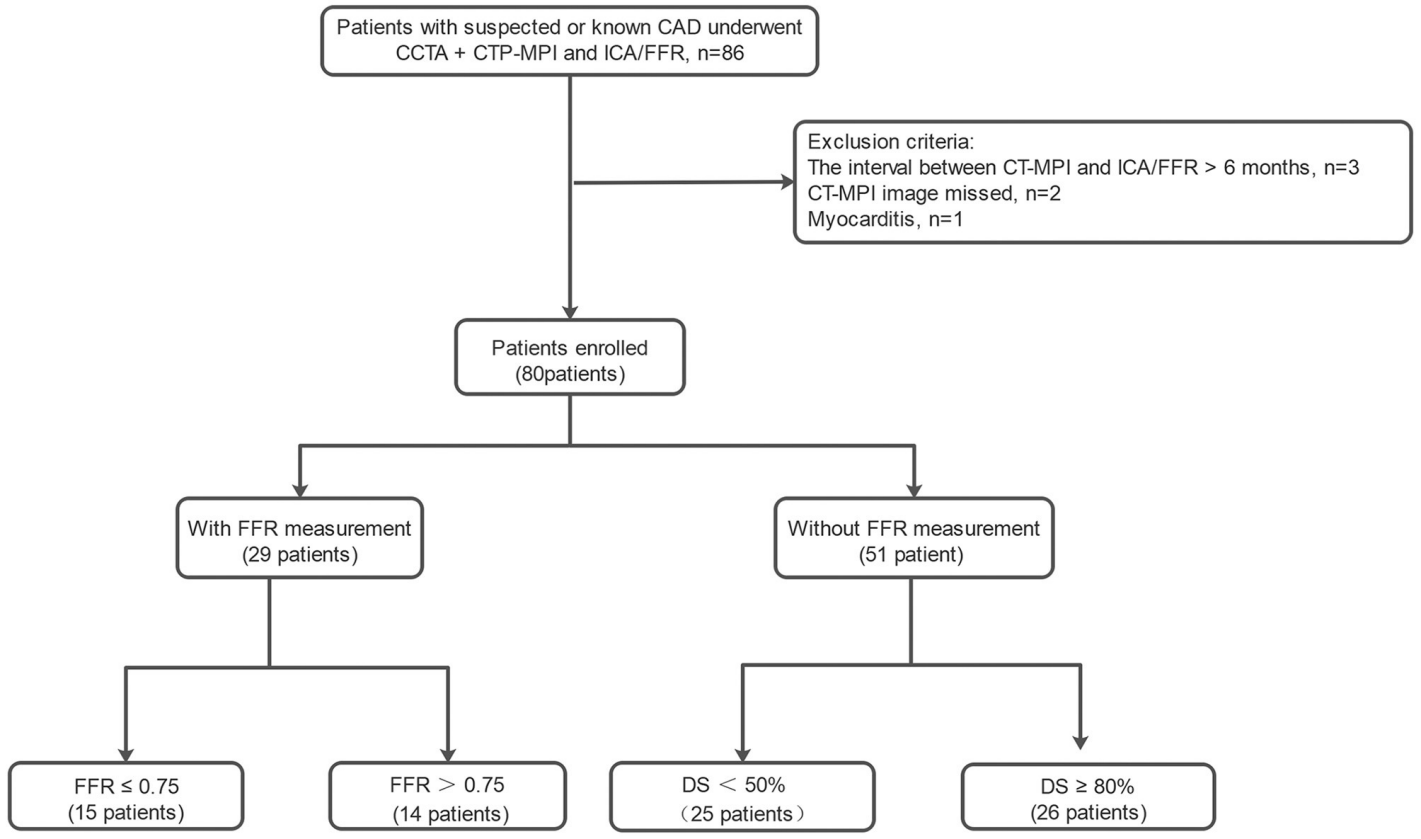
Study flowchart of inclusion and exclusion for the patients. CCTA, coronary computed tomography angiography; CT-MPI, computed tomography myocardial perfusion imaging; DS, diameter stenosis; FFR, fractional flow reserve; ICA, invasive coronary angiography.

A total of 80 consecutive symptomatic patients [age, 58 ± 10 years (37–82 years); 55 men] who completed CT-MPI and invasive coronary angiography (ICA)/fractional flow reserve (FFR) examinations between August 2016 and January 2021 were retrospectively recruited and constituted the validation data set (Figure 1). The median interval between CT-MPI and ICA/FFR examinations was 9 (4–26) days. The inclusion criteria were suspected or known CAD with stable angina or angina-equivalent symptoms and being aged 18 years or older. The exclusion criteria were as follows: (a) acute coronary syndrome or clinical instability, (b) non-ischemic cardiomyopathy, (c) contraindications to adenosine or iodine contrast media, (d) pregnancy, (e) missed image, (f) the interval between CT-MPI and ICA/FFR more than 6 months.

The study was approved by the local institutional ethical committee. All enrolled participants gave written informed consent.

### Sample Size Calculation

According to the diagnostic performance of dynamic CT MPI on the third – generation DSCT scanner reported by Li et al. (12) the vessel-based sensitivity and specificity of hyperemic myocardial blood flow (MBF) for detecting hemodynamically significant stenosis are 96 and 93%, respectively. We assumed the sensitivity and specificity for hyperemic MBF to be equal to 95 and 90%. Based on these assumptions, using a two-sided binomial test, a total of 160 vessel participants were required to achieve 80% efficacy for rejecting the null hypothesis. The target significance level is 0.05. A total of 198 vessels were included, which provided >90% power. The sample size was estimated using Power Analysis and Sample Size software (version 15.0.5; PASS).

### Dynamic CT-MPI and CCTA Protocol

All participants were scanned with a third – generation DSCT scanner (SOMATOM Force; Siemens Healthineers, Germany). A “one-stop” CT-MPI + CCTA protocol integrating calcium score, dynamic CT-MPI, and CCTA was employed. Initially, calcium score scan was performed. Then, dynamic CT-MPI was performed after 3 min of continuous adenosine administration at an infusion rate of 160 μg/kg/min. Iodinated contrast agent (100 mL; Ultravist, 370 mg iodine/mL, Bayer, Germany) was given in a bolus injection at a flow rate of 5.2 mL/s, followed by a 40 mL saline flush. Dynamic CT- MPI image acquisitions were started 4 s after contrast injection was started. The shuttle-mode acquisition technique is used to image the complete left ventricle. By moving the table back and forth after each acquisition, two series of images were collected, which together covered the entire myocardium. Dynamic image acquisition was performed in systole, 250 ms after the R - wave. Depending on heart rate, scans were launched every second or third heart cycle, resulting in a series of 10–15 data samples acquired over a period of ~32 s. The following scan parameters were used: collimation = 192 × 0.6 mm, gantry rotation time = 250 ms, temporal resolution = 66 ms, shuttle-mode z-axis coverage of 105 mm, tube voltage = 70 kV, and automated tube current scaling. CARE kV and CARE dose 4D was used to reduce radiation dose.

Nitroglycerin was given sublingually to all participants 5 min after CT-MPI. Subsequently, a bolus of contrast media was injected into the antecubital vein at a rate of 4–5 ml/s, followed by a 40 mL saline flush using a dual—barrel power injector. Retrospective electrocardiography—triggered sequential acquisition was carried out in all patients undergoing CCTA, with the center of the triggering window set at diastole or systole, depending on the heart rate. With application of automated tube voltage and current modulation, the reference tube current was set as 320 mAs, and the reference tube voltage was set as 100 kV.

### CCTA and CT-MPI Image Processing

The CT-MPI images were reconstructed with CT-MPI software package (VPCT body, Siemens Healthineers) using a dedicated kernel to reduce iodine beam-hardening artifacts (b23f, Qr36). All reconstructed images were transferred to a workstation (Syngo.Via VB10B; Siemens Healthineers) for analysis. Motion correction was performed for all images. Quantification of MBF was performed using a hybrid deconvolution model, as previously reported by Coenen et al. (15). To sample the MBF, the region of interest (ROI) was manually placed on short-axis view on a segment basis according to the American Heart Association (AHA) 17-segment model (16). The ROI was drawn to cover the whole segment without perfusion defect or cover the whole area of suspected perfusion defects within the segment. According to AHA recommendation, individual myocardial segments were assigned to the three major coronary arteries territories and was adjusted for differences in dominance (16). The global MBF was the mean MBF value of all 17 myocardial segments, and regional MBF was calculated as the average MBF value of the myocardial segments it included. Two experienced cardiovascular radiologists who were blinded to the participants' clinical history independently analyzed the CCTA and CT-MPI data. Image quality for each segment was assessed with a 4-point scale as previously described (17).

### ICA and Invasive FFR

All patients underwent ICA with standard methods. All coronary arteries and main branches were evaluated by two interventional cardiologists. During steady-state hyperemia, FFR was measured using a 0.014-inch pressure guidewire (Prime Wire Prestige PLUS, Volcano Corporation). Hyperemia was induced by intravenous infusion of adenosine at 140 mg/kg/min. FFR measurements were performed in intermediate stenosis (defined as a diameter reduction between 30 and 90% on visual assessment). Ischemic lesions were defined as lesions with more than 90% stenosis or intermediate lesions with FFR ≤ 0.80. Non-ischemic lesions were defined as lesions with <30% stenosis or intermediate lesions with FFR > 0.80. Vessels causing ischemia were defined as arteries with one or more ischemic lesions. The most severe stenosis was considered for analysis in the same perfusion territory.

### Statistical Analysis

The Shapiro–Wilk test was used to determine if the data had a normal distribution. Continuous variables were presented as the mean ± standard deviation (SD) or as the median and quartile. ANOVA or Student's *t*-test was used for normally distributed data, and the Mann-Whitney *U*-test was used for non-normally distributed data. Bonferroni correction was applied for multiple comparisons. Categorical variables were presented as the number and proportion. The coefficient of variation (CV, %) of hyperemic MBF in both the volunteer group and patient group were calculated as (SD/mean) × 100%. Bivariate Pearson correlation analyses were performed between the age and hyperemic MBF and between BMI and hyperemic MBF. The normal range for hyperemic MBF was defined as that which would include 95% of the population. On the basis of the normality of data distribution, the normal range was calculated as the mean ± SD × 1.96. Cut-off value was defined as the lower limit of the normal range. Cohen's Kappa statistic was used to compare the MBF derived from CT-MPI with the ICA/FFR in diagnosing myocardial ischemia. Intra-observer and inter-observer agreements of MBF measurements were manually tested for intraclass correlation coefficients and by using Bland–Altman plots. Effective radiation dose was calculated by multiplying the dose-length product by a constant conversion factor (*k* = 0.026 mSv/mGy/cm) (18). A 2-sided p < 0.05 was considered statistically significant. All statistical analyses were performed using an SPSS software package (SPSS 26.0; IBM) and a MedCalc software package (MedCalc 15.2.0).

## Results

### Baseline Characteristics of Healthy Participants and Patients

Details of excluded patients are presented in **Figure 1**. Forty-two vessels from 80 enrolled patients with 30–90% luminal stenosis but without FFR were excluded from the analysis. A total of 198 vessels from 80 enrolled patients were ultimately included for the analysis. As shown in Table 1, the enrolled patients were mainly male and were significantly older and more overweight than the volunteers.

**Table 1:** The demographic, clinical and imaging characteristics of healthy participants and patients.

**Characteristics**	**Healthy participants (*n* = 51)**	**Patients (*n* = 80)**	***P*-value**
Age, years	38 ± 11.5	58 ± 10.1	<0.001^*^
Male gender (%)	16/51 (31)	55/80 (69)	<0.001^*^
Body mass index (kg/m^2^)	22.7 ± 2.6	25.9 ± 3.0	<0.001^*^
**Coronary risk factors**			
Hypertension (%)	-	55/80 (69)	
Dyslipidemia (%)	-	73/80 (91)	
Diabetes (%)	-	19/80 (24)	
Smoking (%)	-	42/80 (53)	
Family history of CAD (%)	4/51 (8)	16/80 (20)	0.059
**Symptoms**			
Typical angina (%)	-	38/80 (48)	
Atypical angina (%)	-	35/80 (44)	
Non-cardiac chest pain (%)	-	7/80 (9)	
**Distribution of coronary artery**			
Right dominance (%)	49/51 (96)	77/80 (96)	
Left dominance (%)	1/51 (2)	2/80 (3)	
Balanced (%)	1/51 (2)	1/80 (1)	
**Stenosis extent (diameter stenosis, %)**			
<50 (%)	-	121/198 (61)	
50–90 (%)	-	36/198 (18)	
≥90 (%)	-	41/198 (21)	
Vessels with ischemic lesion	-	67/198 (34)	
Left anterior descending artery (%)	-	33/198 (17)	
Left circumflex coronary artery (%)	-	15/198 (8)	
Right coronary artery (%)	-	19/198 (10)	
**Serum markers**			
Cholesterol (mmol/L)	4.14 (3.45–4.86)	3.39 (2.99–4.25)	0.006^*^
HDL-C (mmol/L)	1.44 ± 0.30	1.11 ± 0.25	<0.001^*^
LDL-C (mmol/L)	2.24 (1.80–2.99)	1.86 (1.53–2.61)	0.119
Triglyceride (mmol/L)	0.86 (0.59–1.24)	1.31 (0.90–1.78)	<0.001^*^
Fasting plasma glucose (mmol/L)	4.83 (4.58–5.06)	5.13 (4.61–5.58)	0.034^*^
Creatinine (μmol/L)	63 (55–77)	68 (57–77)	0.254
**Hemodynamic changes in CT-MPI scan protocol**			
Rest heart rate (beats/min)	77 ± 12	72 ± 12	0.234
Stress heart rate (beats/min)	100 (89–109)	91 (78–103)	0.007^*^
Δ Heart rate (beats/min)	29 (20–32)	23 (17–29)	0.003^*^
Rest SBP (mmHg)	120 ± 13	136 ± 14	<0.001^*^
Rest DBP (mmHg)	73 ± 8	81 ± 10	<0.001^*^
Hyperemic MBF (ml/100 ml/min)	164 ± 24	123 ± 26	<0.001^*^

*Measurement data are means ± standard deviations, or medians, with interquartile ranges in parentheses. Categorial data are numbers of patients or vessels, with percentages in parentheses. CAD, coronary artery disease; HDL-C, high density lipoprotein cholesterol; LDL-C, low density lipoprotein cholesterol; Δ Heart rate, Stress heart rate -Rest heart rate; CCTA, coronary computed tomography angiography; CT-MPI, computed tomography myocardial perfusion imaging; SBP, systolic pressure; DBP, diastolic pressure; MBF, myocardial blood flow*.

* *P-values < 0.05 refers to results of Student's t-tests for normal distribution data, Mann-Whitney U-tests for non-normal distribution data, and Chi-square test for Categorial data*.

### Image Quality and Radiation Dose of CCTA and CT-MPI

All participants tolerated the procedures well. Supplementary Table E1 shows the image quality of CT-MPI. The proportion of non-diagnostic segments with poor image quality for the volunteer group and the patient group was 0.1 and 0.3%, respectively. The radiation doses of CCTA in healthy participants and patients were 11.1 (8.8 – 13.9) mSv and 13.6 (11.2 – 16.1) mSv, respectively. The radiation doses of dynamic stress CT-MPI in healthy participants and patients were 5.9 (4.6 – 9.0) mSv and 7.1 (5.6 – 9.0) mSv, respectively.

### Hyperemic MBF of Healthy Participants

**Figure 2:**
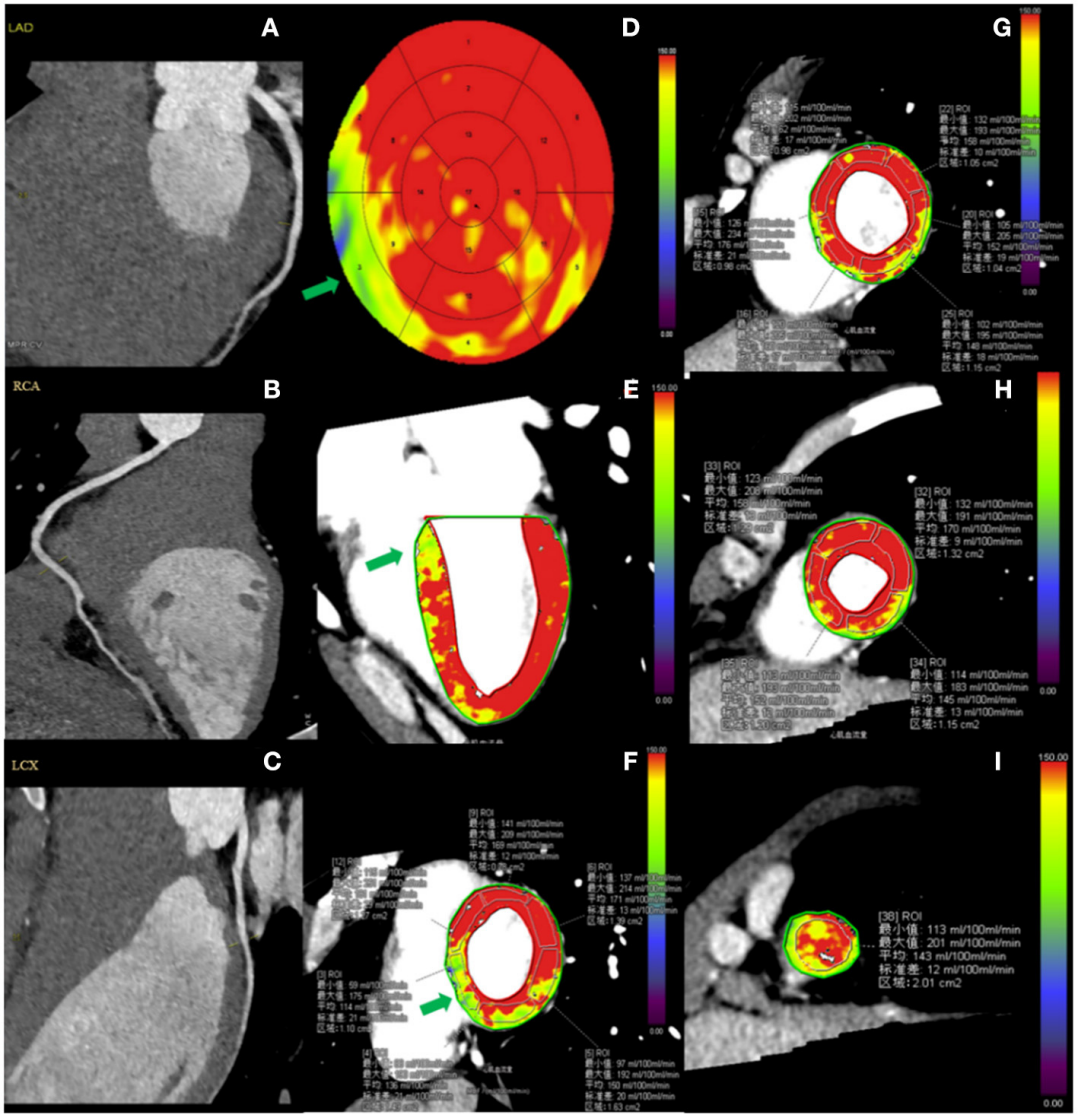
Case example of CCTA and hyperemic MBF distribution in a healthy participant. A 29-year-old male healthy volunteer. **(A–C)** Resting CCTA shows no calcified plaque or stenosis in the coronary arteries. **(D–I)** Stress CT-MPI during adenosine infusion showed normal myocardial perfusion, as shown by relatively homogeneous color-coded images in the bull's eye diagram **(D)** and left ventricular long-axis view **(E)** and short-axis views **(F–I)**. **(D–F)** The green arrow shows that the hyperemic MBF of basal-septum was lower than that of other segments. **(F,G)** ROI was manually placed in each myocardial segment as large as possible, excluding a 1 mm endocardial and epicardial borders to avoid image artifacts. CCTA, coronary computed tomography angiography; CT-MPI, computed tomography myocardial perfusion imaging; MBF, myocardial blood flow; ROI, Region of interest.

In healthy participants, the global hyperemic MBF was 164 ± 24 ml/100 ml/min (range 114–228 ml/100 ml/min; CV, 14.6%). Hyperemic MBF values were homogeneously distributed among myocardial regions (all *P* > 0.05, Table 2), but not among 17 myocardial segments (*P* = 0.006; Table 3). The hyperemic MBF of the basal inferoseptal (segment 3) was significantly lower than that of the other segments. Representative case examples are illustrated in Figure 2. There was no significant difference in the MBF between other myocardial segments.

**Table 2:** Regional distribution of hyperemic MBF.

	**Hyperemic MBF**	***P*-value**
	**(ml/100 ml/min)**	
**Vessel territories**		0.399
LAD	167 ± 24 (116–233)	
RCA	164 ± 24 (108–225)	
LCX	160 ± 25 (113–226)	
**Regions**		0.606
Anterior	165 ± 24 (117–218)	
Septum	165 ± 22 (115–243)	
Inferior	167 ± 28 (107–225)	
Lateral	160 ± 25 (108–225)	
**Axial level**		0.478
Basal	161 ± 23 (113–227)	
Middle	167 ± 25 (112–238)	
Apical	164 ± 25 (119–223)	

*Data are means ± standard deviations; numbers in parentheses are ranges. The AHA 17 segments model of left ventricular were assigned to 3 major coronary arteries territories: LAD (segments 1, 2, 7, 8, 13, 14, and 17), RCA (segments 3, 4, 9, 10, and 15), LCX (segments 5, 6, 11, 12, and 16); four myocardial regions: anterior (Segments 1, 7, 13, 17), septum (Segments 2, 3, 8, 9, and 14), inferior (segments 4, 10, and 15), and lateral (segments 5, 6, 11, 12, and 16); and basal (segment 1–6), middle (segment 7–12), and apical (segment 13–16) for the analysis. MBF, myocardial blood flow; LAD, left anterior descending coronary artery; LCX, left circumflex coronary artery; RCA, right coronary artery*.

*P-values were derived from one-way ANOVA*.

**Table 3:** Hyperemic MBF of 17 segments.

**Segments**	**Hyperemic MBF (ml/100 ml/min)**
Basal anterior^a^	169 ± 27 (117–234)
Basal anteroseptal^a^	164 ± 25 (114–243)
Basal inferoseptal^b^	145 ± 20 (108–207)
Basal inferior^a^	169 ± 27 (108–221)
Basal inferolateral^ab^	158 ± 26 (94–237)
Basal anterolateral^a^	164 ± 27 (106–231)
Mid anterior^a^	169 ± 28 (104–229)
Mid anteroseptal^a^	177 ± 27 (114–272)
Mid inferoseptal^a^	171 ± 26 (121–250)
Mid inferior^a^	167 ± 29 (100–228)
Mid inferolateral^ab^	161 ± 27 (107–208)
Mid anterolateral^ab^	159 ± 25 (108–222)
Apical anterior^ab^	163 ± 24 (120–223)
Apical septal^a^	169 ± 26 (113–243)
Apical inferior^a^	165 ± 31 (105–233)
Apical lateral^ab^	160 ± 26 (112–209)
Apex^ab^	158 ± 26 (108–205)

*Data are means ± standard deviations, numbers in parentheses are ranges. P-values were calculated with a one-way ANOVA and post-hoc Bonferroni to assess significance. MBF, myocardial blood flow; Min, minimum value; Max, maximum value*.

*Letter a and b indicate significant difference between segmental MBF values (p < 0.05), and letter ab represents the value has no significant difference with a and b (p > 0.05)*.

### Effect of Age, Gender, and BMI on Hyperemic MBF of Healthy Subjects

**Figure 3:**
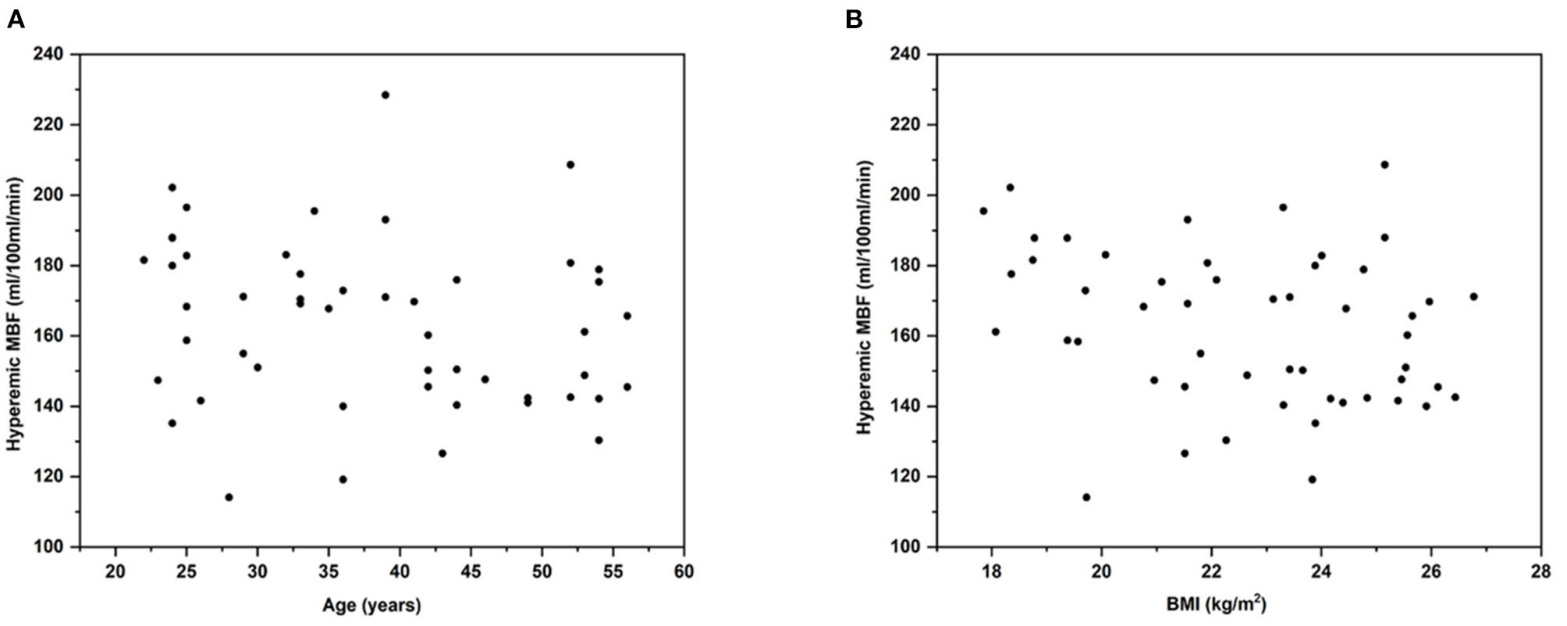
Age and BMI vs. hyperemic MBF in healthy volunteers. Pearson correlation analysis of age and BMI vs. hyperemic MBF. Scatterplot shows the relationship between **(A)** age and hyperemic MBF (y = 181.76 - 0.468x; *r* = −0.22; *R*^2^ = 0.048; *P* = 0.1242) and **(B)** BMI and hyperemic MBF (y = 214.78 - 2.300x; *r* = −0.27; *R*^2^ = 0.072; *P* = 0.0570). This study could not demonstrate significant relationship between age, BMI and hyperemic MBF. BMI, body mass index; MBF, myocardial blood flow.

The hyperemic MBF in females appeared to be higher than that of males (168 ± 24 ml/100 ml/min vs. 155 ± 20 ml/100 ml/min, *P* = 0.082), but the difference was not statistically significant. No significant correlation between hyperemia MBF and age (Figure 3A) was observed. Similarly, no significant correlation between hyperemia MBF and body mass index (BMI) was observed normal volunteers (Figure 3B).

### Normal Reference Range of Hyperemic MBF

According to the Shapiro-Wilk test, at the per-patient, per-vessel and per-segment levels, MBF were all normally distributed. In the per-vessel analysis, the regional MBF was 164 ± 24 ml/100 ml/min (range 108–233 ml/100 ml/min). The reference range of the regional MBF was 116–211 ml/100 ml/min.

### Hyperemic MBF of Patients

**Figure 4:**
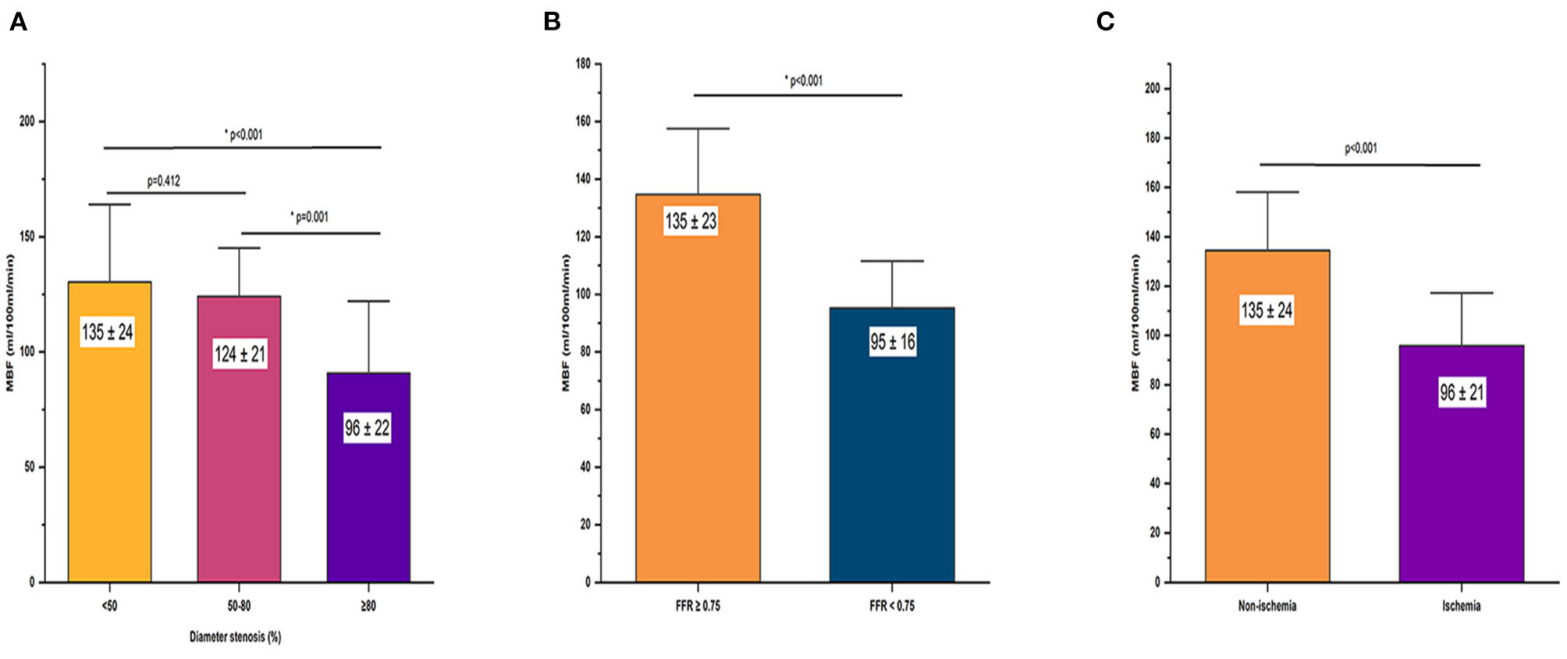
Relationship between hyperemic MBF and severity of stenosis. Bar plots shows **(A)** hyperemic MBF for vessel territories with server stenosis (DS > 90%) was significantly lower compared with vessel territories without significant stenosis (DS < 30%) and vessel territories with intermediate stenosis (DS 30–90%) at ICA. **(B)** Hyperemic MBF of non-ischemic myocardium was significantly lower than ischemic myocardium defined by ICA/FFR. **(C)** Hyperemic MBF was significantly lower in vessels with FFR of ≤ 0.80 compared with that in vessels with FFR > 0.80. DS, diameter stenosis; FFR, fractional flow reserve; ICA, invasive coronary angiography; MBF, myocardial blood flow.

Patients had lower global hyperemic MBF values (123 ± 26 ml/100 ml/min) and greater heterogeneity (CV, 21%) than healthy volunteers. In per-vessel analysis, the regional MBF of non-ischemic vessels was significantly lower than that of healthy people (135 ± 24 ml/100 ml/min vs. 164 ± 24 ml/100 ml/min, *p* < 0.001) and higher than that of ischemic vessels (96 ± 201 ml/100 ml/min vs. 135 ± 24 ml/100 ml/min, *p* < 0.001). Details of the relationship between the MBF and stenosis severity were shown in Figure 4.

### Diagnostic Performance of the Calculated Normal MBF Range for Predicting Ischemic Stenosis

**Figure 5:**
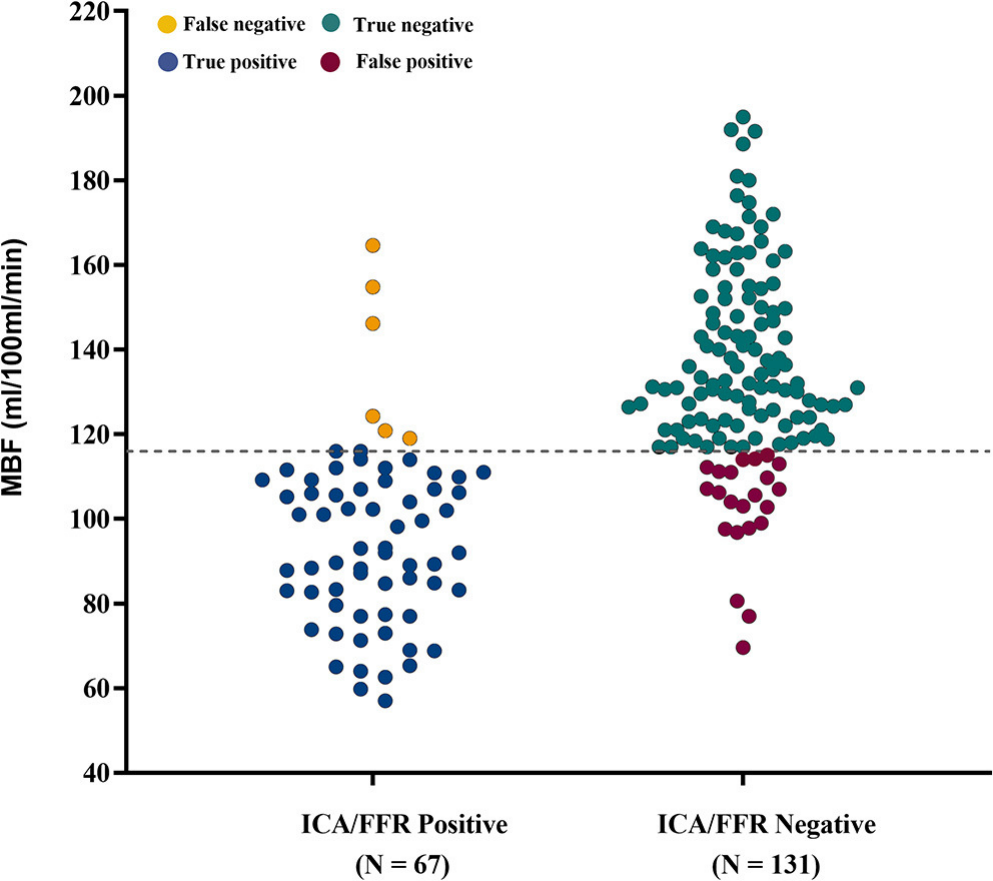
Diagnostic classification by CT-MPI and ICA/FFR. Scatter dot plot of all observations in per-vessel analysis. Gray dashed line represents the cutoff value of MBF of 116 ml/100 ml/min. Yellow dots represent false negatives (*n* = 6), blue dots represent true positives (*n* = 61), green dots represent true negatives (*n* = 109), and red dots represent false positives (*n* = 22). FFR, fractional flow reserve; ICA, invasive coronary angiography; MBF, myocardial blood flow.

**Figure 6:**
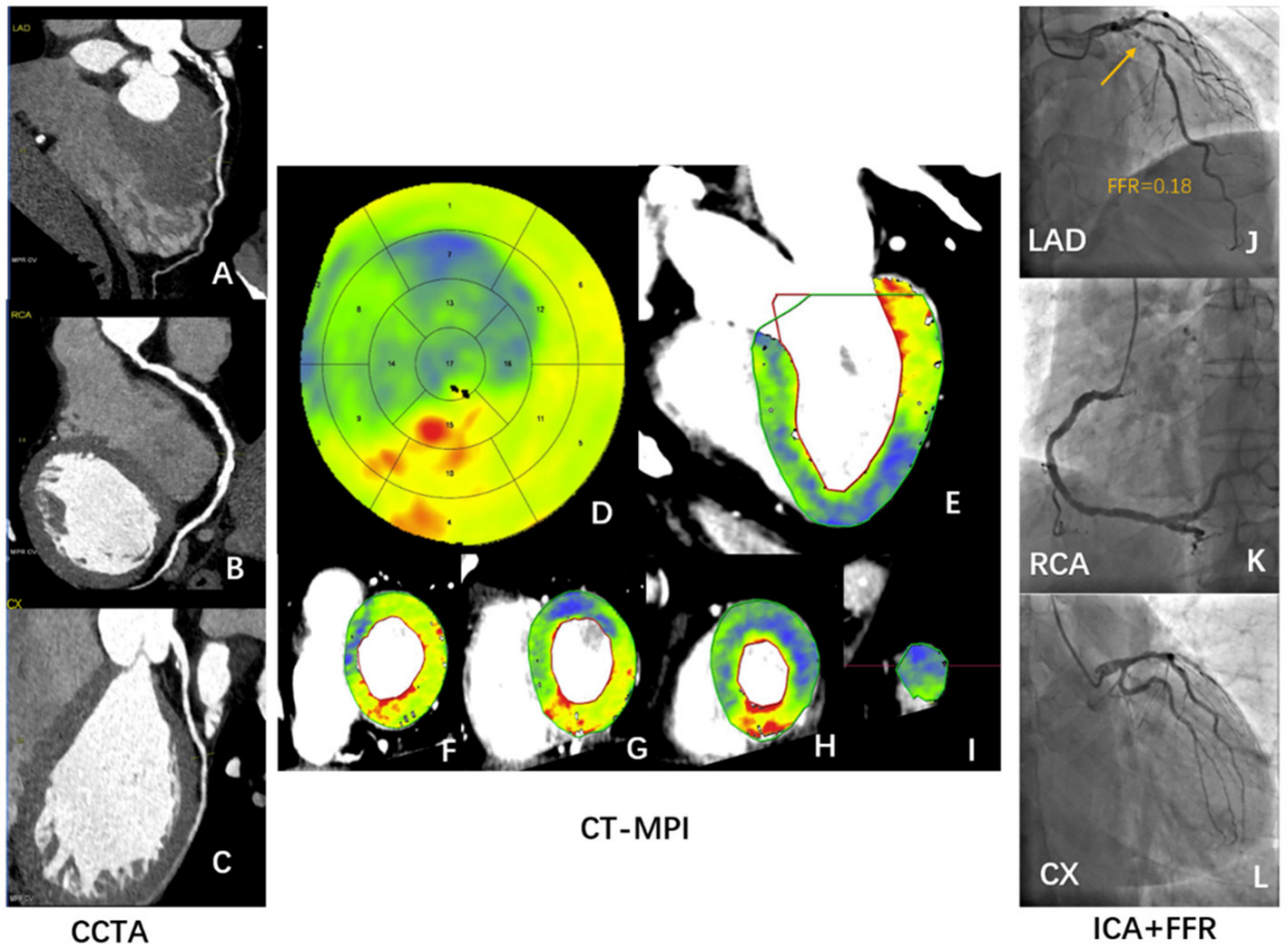
Case illustrating hyperemic MBF can identify ischemic stenosis confirmed by ICA/FFR. A 56-year-old man who presented with a history of hypertension, current smoking, symptomatic for suspected angina, and a recent inconclusive 24 h' DCG. **(A–C)** Rest CCTA shows severe stenosis of distal LAD **(A)** and multiple mild stenosis of RCA **(B)**. **(D–I)** Dynamic stress CT-MPI bull's eye diagram **(D)**, long axis view **(E)**, and short axis view **(F–I)** all show severe induced perfusion defects in the anterior wall, septum, and apical wall of left ventricle. The regional hyperemic MBF of LAD, RCA, LCX are 77 ml/100 mml/min, 126 ml/100 ml/min, and 107 ml/100 ml/min, respectively. **(J–L)** ICA shows severe proximal LAD stenosis **(J)** with positive invasive FFR **(J)**. ICA shows multiple mild stenosis in RCA **(K)** and no stenosis of LCX **(L)**. CCTA, coronary computed tomography angiography; CT-MPI, computed tomography myocardial perfusion imaging; DCG, dynamic cardiogram; FFR, fractional flow reserve; ICA, invasive coronary angiography; LAD, left anterior descending coronary artery; LCX, left circumflex coronary artery; MBF, myocardial blood flow; RCA, right coronary artery.

Of the 198 vessels, 67 vessels were diagnosed as ischemic lesions by ICA/FFR. MBF with a cutoff value of <116 ml/100 ml/min showed good diagnostic performance in diagnosing ischemic lesions. The diagnostic accuracy, sensitivity, specificity, PPV, NPV and area under the curve (AUC) were 85.9% (170/198), 91.0% (61/67), 83.2% (109/131), 73.5% (61/83), 94.8% (109/115), and 0.871 [(CI, 0.817 – 0.926), *p* < 0.001], respectively (Figure 5). With a cutoff value of 116 ml/100 ml/min, CT-MPI showed good consistency with ICA/FFR, with a Cohen's kappa statistic of 0.7016 (95% CI, 0.6009 – 0.8023). A representative case is illustrated in Figure 6.

### Reproducibility of MBF Measurements

**Figure 7:**
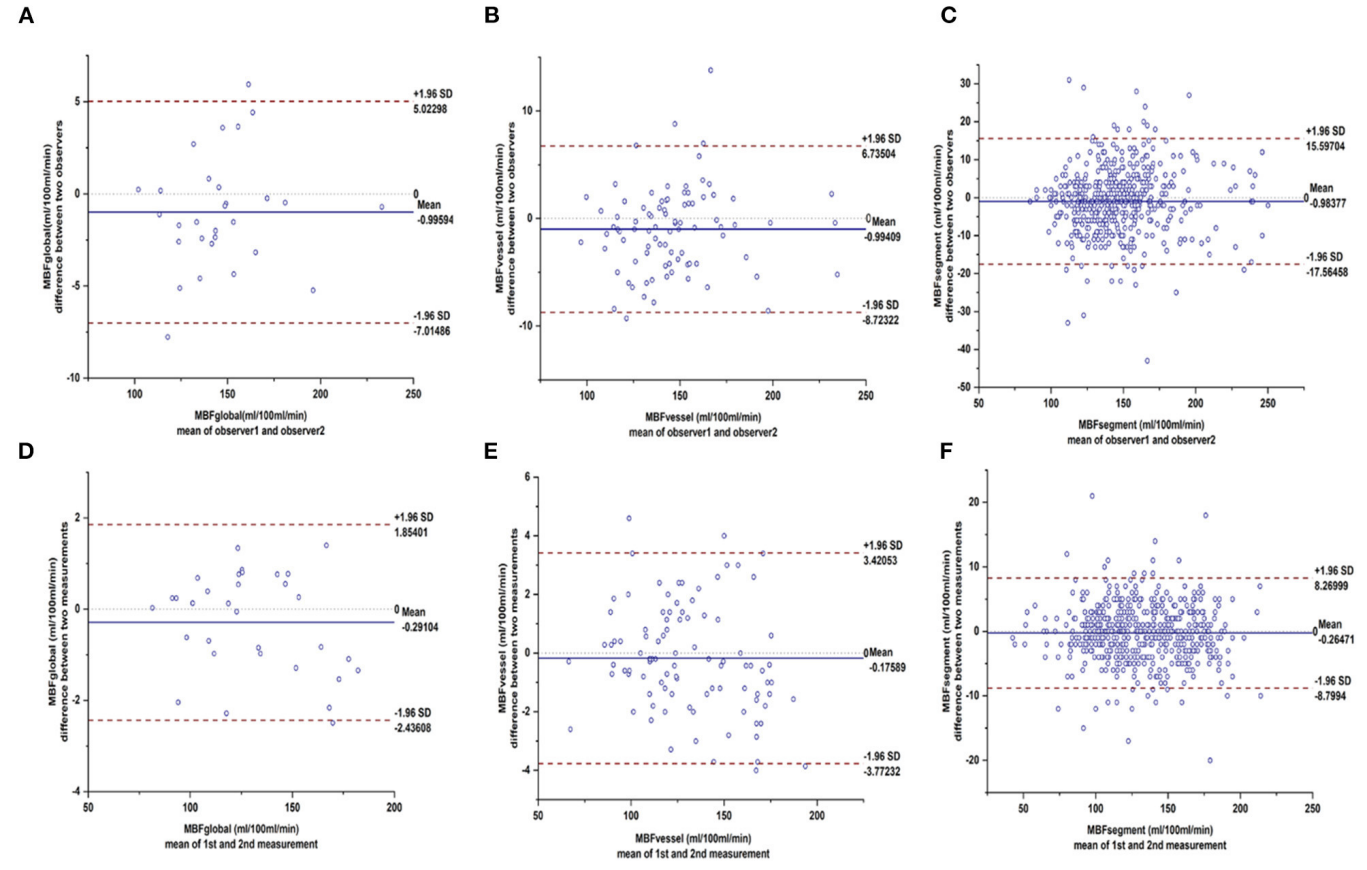
Bland–Altman plots showed excellent inter- and intra-observer agreements of hyperemic MBF. Bland-Altman plots shows the 95% limits of agreement and the mean differences for intra-observer reliability of MBF in **(A)** segmental level, **(B)** vessel level, **(C)** individual level and inter-observer reliability of MBF in **(D)** segmental level, **(E)** vessel level, **(F)** individual level. MBF, myocardial blood flow.

As shown in Figure 7, there was excellent intra- and inter- observer reproducibility of manual MBF measurements. The intraclass correlation coefficients for the global MBF, regional MBF, and segmental MBF were 0.996, 0.991, and 0.990, respectively (all *p* < 0.001). The interclass correlation coefficients for the global MBF, regional MBF, and segmental MBF were 0.993, 0.989, and 0.955, respectively (all *p* < 0.001).

## Discussion

The main findings of the present study can be summarized as follows: (a) the hyperemic MBF was heterogeneous in both healthy individuals and patients; (b) patients had lower hyperemic MBF and greater heterogeneity than healthy subjects; and (c) the normal range hyperemic MBF with third—generation DSCT was 116–211 ml/100 ml/min. The normal range of hyperemic MBF defined in healthy subjects can help in the diagnosis of myocardial ischemia defined by ICA/FFR.

### Cut-Off Values of Hyperemic MBF Assessed by CT

The lower limit of the normal range of MBF derived from healthy volunteers can help identify ischemia, and the diagnostic efficacy is comparable to that of previous studies (Table 4). Both the optimal cutoff of MBF and the MBF of the remote myocardium reported by Bamberg et al. (5), Greif et al. (19), and Rossi et al. (20) was significantly lower, which may be due to the fact that these studies included patients with MI and a history of revascularization. Studies excluding patients with a history of MI and a history of revascularization reported a cutoff value of MBF ranging from 89.5 to 106 ml/100 ml/min, and the MBF of the remote myocardium ranged between 116 ml/100 ml/min and 169 ml/100 ml/min (10, 12, 15, 21–25), which are consistent with the present study. Obviously, the severity of myocardial ischemia in patients affects the optimal cutoff value of MBF. In addition, coronary microvascular dysfunction (CMVD) may be another main pathophysiological factor leading to decreased MBF.

**Table 4:** Quantitative myocardial blood flow and ischemic cut-off values of stress MBF assessed by CT-MPI.

**References**	**Equipment**	**Normal MBF** **(ml/100 ml/min)**	**Ischemic MBF** **(ml/100 ml/min)**	**Cutoff value** **(ml/100 ml/min)**	**Reference standard**	**Diagnostic accuracy**	**Sen**	**Spe**	**PPV**	**NPV**	**Objects** **(MI, revascularization)**
Bamberg et al. (5)	2nd DSCT	104.8 ± 34.0	73.2 ± 26.0	75	FFR ≤ 0.75 + ICA ≥ 85% DS	N/A	N/A	N/A	N/A	N/A	Yes, yes
Greif et al. (19)	2nd DSCT	122.7 ± 34.0	78.7 ± 26.1	75	FFR ≤ 0.80 + ICA ≥ 90% DS	78.2	95.1	74.0	49.3	98.3	Yes, yes
Rossi et al. (20)	2nd DSCT	109	62	78	FFR ≤ 0.75 + ICA ≥ 90% DS	90	88	90	77	95	No, yes
Kono et al. (21)	2nd DSCT	116.3 ± 27	25.6 ± 22.5	103.1	FFR ≤ 0.80	68.1	88.9	47.8	62.5	81.5	No, no
Wichmann et al. (25)	2nd DSCT	140 ± 38.4	80.7 ± 33.7	103	CCTA ≥ 50% DS	62.9	82.4	80.5	60.1	92.8	No, no
Li et al. (12)	3st DSCT	169 ± 34	75 ± 20	99	FFR ≤ 0.80 + ICA ≥ 90% DS	94	96	93	92	96	No, no
Li et al. (22)	3st DSCT	133	78	89.5	FFR ≤ 0.80	90.5	84.3	97.7	97.7	84.3	No, no
Coenen et al. (15)	3st DSCT	108	79	91	MRI	68	75	61	63	73	No, no
Rossi et al. (24)	2nd DSCT	161	92 (74–109)	106	FFR ≤ 0.80 + ICA ≥ 80% DS	N/A	75	88.3	68.3	91.3	No, no
Pontone et al. (23)	Revolution CT	130 ± 46	96 ± 32	101	FFR ≤ 0.80 + ICA ≥ 80% DS	78	86	75	60	93	No, no
Yi et al. (10)	3st DSCT	147.5 ± 25.6	91.5 ± 29.9	N/A	FFR ≤ 0.80 + ICA ≥ 80% DS	92	83	99	98	90	No, no
Current study	3st DSCT	164 ± 24	96 ± 21	116	FFR ≤ 0.75 + ICA ≥ 80% DS	85.9	91.0	83.2	94.8	73.5	No, no

*CCTA, Coronary computed tomography angiography; CT-MPI, computed tomography myocardial perfusion imaging; DS, diameter stenosis; DSCT, dual-source CT scanner; FFR, fractional flow reserve; ICA, interventional coronary angiography; MBF, myocardial blood flow; MI, myocardial infarction; NPV, negative predictive value; N/A, not reported; PPV, positive predictive value; Sen, sensitivity; Spe, specificity*.

### Aging, Sex, Body Mass Index, and Hyperemic Myocardial Blood Flow

The present study showed the hyperemic MBF was not significantly different between males and females, and similar findings were reported in previous studies using either adenosine or dipyridamole infusion in normal subjects (7, 26). In normal volunteers aged 22–59 years, this study did not find that hyperemic MBF declines with increasing age. According to Chareonthaitawee et al. (7) and Uren et al. (26), there was a significant decrease in the hyperemic MBF among healthy subjects aged over 65 or 70 years. These changes are likely to result from various factors associated with aging including endothelial dysfunction. Whether MBF reduction in the elderly is a pathological change due to possible CMVD is worth considering. Studies have shown that the increase in BMI is accompanied by endothelial dysfunction (27). Obesity is one of the risk factors for atherosclerosis (28). To define the range of true normal human myocardial perfusion as physiological guides in clinical studies or management, we selected young volunteers who were <60 years old and were not obese.

### Spatial Heterogeneity of Myocardial Blood Flow

Describing the distribution of MBF in normal humans may assist us in better recognizing and understanding ischemia. The present study showed that the hyperemic MBF of the basal septum was lower than that of other segments, while the hyperemic MBF of the middle septum was higher than that of other segments. A similar phenomenon in adenosine-stress dynamic CT-MPI has previously been reported (6, 29). This spatial heterogeneity may have occurred due to the following reasons: (a) At the interventricular junction area, the membranous part of the septum is mainly formed by fibrous tissue, and the middle and apical part of the septum are mainly composed of muscular tissues. The difference in tissue composition and capillary distribution may be the basis of the spatial heterogeneity of the MBF in the septum. (b) Beam-hardening and motion artifacts may have substantial impact on image interpretation. Clinicians should be fully aware of the spatial heterogeneity of the MBF distribution as described above, to avoid unnecessary economic burden and psychological pressure on patients due to incorrect interpretation of the results of myocardial perfusion.

### Limitations

Our study has several limitations. Firstly, this study was a small, single-center study and all images were obtained by third—generation DSCT scanner. The MBF value might vary from a different CT scanner, different MBF calculation methods, different pharmacological stressors and different ethnicities. The normal reference value range of MBF reported by our study may only be applicable to the scenario with similar examination and postprocess protocols, and MBF calculation methods. Secondly, all participants were Chinese, our findings not be extrapolated to other ethnicities or populations. Therefore, further studies are needed to establish a normal-value database for MBF and the present study contributes to that effort. Thirdly, the MBF derived from CT-MPI does not represent the true blood flow of the myocardium. Specifically, in CT imaging iodinated contrast agents have an extraction fraction in the range of 10–40% depending on the actual flow. This is a major confounder of MBF quantification as compared to other more realistic methods and leads to a systematic underestimation of MBF in CTP imaging. In addition, the limited temporal sampling rate of the shuttle mode and the hybrid deconvolution also contribute to the underestimation of MBF (30–33). Finally, we verified the diagnostic value of the MBF optimal cutoff value in patients with non-CAD and with severe ischemia, which might result in an overestimation of its diagnostic performance.

## Conclusion

This study provides the normal range of hyperemic MBF in healthy subjects using a third—generation DSCT scanner. Recognizing hyperemic MBF in healthy subjects helps better understand myocardial ischemia in CAD patients. Developing a dataset for CT-MPI in a large-sample, multi-center, multi-ethnic, multi-vendor sources study in the future is challenging but promising.

## Data Availability Statement

The original contributions presented in the study are included in the article/Supplementary Material, further inquiries can be directed to the corresponding authors.

## Ethics Statement

The studies involving human participants were reviewed and approved by Ethics Committee on Scientific Research of Shandong University Qilu Hospital [KYLL-2016-336]. The patients/participants provided their written informed consent to participate in this study. Written informed consent was obtained from the individual(s) for the publication of any potentially identifiable images or data included in this article.

## Author Contributions

MZ and PZ: conceived and designed study and performed critical revision of the manuscript. LL: conceived and designed study, analyzed data, prepared first draft, and revised subsequent drafts. JP, XL, WY, CG, PL, and MD: assisted in acquisition of CT imaging and contributed to analysis of data. XL, WY, and MD: assisted in acquisition of ICA/FFR data and contributed to analysis of data. DL, YL, and DY: designed and implemented CCTA + CT-MPI protocols, assisted in acquisition of imaging, and analysis of data. YH and JS: contribution to analysis of CCTA and CT-MPI data. All authors contributed to the article and approved the submitted version.

## Funding

This work was supported by the National Key Research and Development Program of China [2016YFC1300302] and the Fundamental Research Funds of Shandong University [2018JC009].

## Conflict of Interest

The authors declare that the research was conducted in the absence of any commercial or financial relationships that could be construed as a potential conflict of interest.

## Publisher's Note

All claims expressed in this article are solely those of the authors and do not necessarily represent those of their affiliated organizations, or those of the publisher, the editors and the reviewers. Any product that may be evaluated in this article, or claim that may be made by its manufacturer, is not guaranteed or endorsed by the publisher.
