# Supplementary material for: Knowledge of Hyperemic Myocardial Blood Flow in Healthy Subjects Helps Identify Myocardial Ischemia in Patients With Coronary Artery Disease
Source: Front Cardiovasc Med. 2022 Feb 3;9:817911. doi: 10.3389/fcvm.2022.817911 (PMC8850642; doi:10.3389/fcvm.2022.817911)
Supplement: Supplementary file 1 [file Data_Sheet_1.PDF]

Table E1. Qualitative Assessment of CT-MPI Image Quality.

Participants	Myocardial segments	Score 4	Score 3	Score 2	Score 1
Volunteers	867	1 (0.1)	9 (1.0)	121 (14.0)	736 (84.9)
Patients	1360	4 (0.3)	22 (1.6)	199 (14.6)	1135 (83.5)

Data are numbers of segments, with percentages in parentheses. CT-MPI, computed tomography myocardial perfusion imaging; score 4, poor, could be an artifact or poor image quality; score 3, moderate, probably an artifact and less likely to be a perfusion defect; score 2, good, probably a defect, good image quality with no or minor artifacts; score 1, excellent, no artifacts.
